# Cause rare de pleurésie hémorragique: descellement d’une vis d’ostéosynthèse

**DOI:** 10.11604/pamj.2020.37.43.13236

**Published:** 2020-09-10

**Authors:** Soumia Fdil, Leila Achachi, Mohammed Raoufi, Laila Herrak, Mustapha El Ftouh

**Affiliations:** 1 Service de Pneumologie, Faculté de Médecine et de Pharmacie, Université Mohammed V, CHU Ibn Sina, Rabat, Maroc

**Keywords:** Pleurésie, hémorragique, descellement de vis, Pleurisy, haemorrhagic, screw loosening

## Abstract

L’apparition de pleurésie hémorragique chez un malade myélomateux est en rapport le plus souvent avec une cause non spécifique de la maladie. La localisation myélomateuse pleurale est rare mais elle doit être éliminée. Nous rapportant le cas d’une cause rare de pleurésie hémorragique sur descellement de vis d’ostéosynthèse chez une patiente de 55 ans, suivie pour myélome multiples depuis 2012. Un diagnostic retenu sur l’aspect hémorragique du liquide pleural, les données de la tomodensitométrie (TDM) thoracique qui a mis en évidence le descellement d’une vis à gauche sur le matériel d’ostéosynthèse avec embout situé en pré vertébral au niveau de l’épanchement pleural, et sur le bilan étiologique qui est revenu négatif.

## Abstract (en)

The occurrence of haemorrhagic pleurisy in patients with myeloma is most often related to a non-specific cause. Pleural myeloma is rare but this diagnosis should be excluded. We here report a rare case of haemorrhagic pleurisy due to the loosening of an osteosynthesis screw. The study involved a 55-year-old female patient who had been followed up for multiple myeloma since 2012. Diagnosis was confirmed by the presence of haemorrhagic pleural effusion, by chest computerized tomography scan which showed the loosening of left-sided osteosynthesis screw with tip located in the prevertebral region at the level of the pleural effusion and by negative etiological assesment.

## Introduction

La survenue d’un épanchement pleurale hémorragique spontané au cours de l’évolution d’un myélome nécessite en premier à éliminer une atteinte plasmocytaire pleurale, malgré son incidence rare, une embolie pulmonaire, une pathologie infectieuse notamment la tuberculose, une amylose ou une autre pathologie tumorale. Nous rapportons une cause rare qui est le descellement d’une vis d’ostéosynthèse rachidienne chez une patiente suivie pour myélome.

## Patient et observation

**Figure 1:**
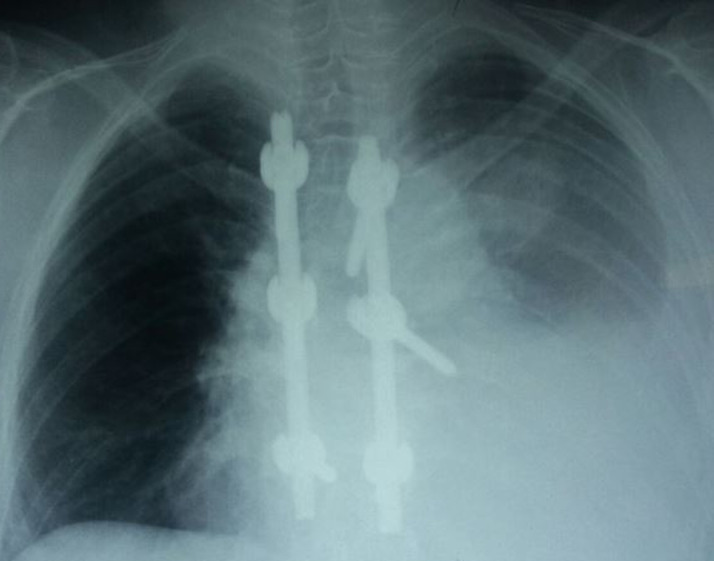
radiographie thoracique de face: épanchement pleural gauche, des images lacunaires au niveau de l’omoplate et les clavicules avec matériel d’ostéosynthèse au niveau du rachis dorsal

**Figure 2:**
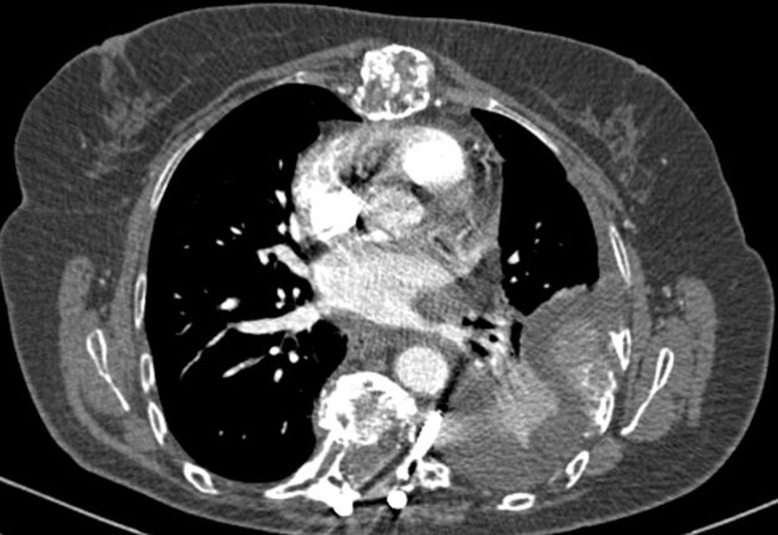
TDM thoracique coupe axiale: descellement d’une vis à gauche sur le matériel d’ostéosynthèse avec embout situé en pré vertébral au niveau de l’épanchement pleural

IL s’agit d’une patiente âgée de 55 ans, suivie pour myélome multiple à chaine légère KAPPA depuis 2012, ayant bénéficié de six cures de chimiothérapie à base de cyclophosphamide, thalidomide, dexaméthasone et radiothérapie, traitement achevé en 2014 et opérée en 2012 aussi pour plasmocytome médullaire avec mise en place d’un matériel d’ostéosynthèse au niveau de D7-D9. L’évolution était marquée par l’apparition d’un plasmocytome frontale pour lequel était remise sous le même protocole de chimiothérapie avec relais par acide zolédronique. La patiente est admise pour une dyspnée d’installation progressive associée à une douleur basithoracique gauche sans notion de traumatisme. L’examen clinique trouve un syndrome d’épanchement liquidien gauche. La radiographie du thorax révélait un épanchement pleural gauche de moyenne abondance, des images lacunaires au niveau de l’omoplate et les clavicules avec matériel d’ostéosynthèse au niveau du rachis dorsal (Figure 1) avec écho-cœur normale. La ponction pleurale trouve un liquide hémorragique non coagulable, l’étude cytologique et chimique montrait des hématies à 2523000 éléments/l, le dosage de l’hématocrite n’a pas été réalisé, des leucocytes à 1236 éléments/l avec prédominance lymphocytaire à 86%, sans cellule malignes, exsudatif avec un taux de protide à 55g/l, et un taux de glucose à 1,02g/l, l’EPP au niveau du liquide pleural n’objective pas de bande mono ou oligo clonal ni zone gamma, la biopsie pleurale révèle un remaniement inflammatoire non spécifique. Les recherches de bacilles acido-alcoolo-résistant dans le liquide pleural et dans les expectorations sont revenues négatives. Un angioscanner thoracique a éliminé la présence d’embolie pulmonaire, et met en évidence le descellement d’une vis à gauche sur le matériel d’ostéosynthèse avec embout situé en pré vertébral au niveau de l’épanchement pleural, en plus de plusieurs lésions osseuses costales, vertébrales et sternale (Figure 2). Une intervention chirurgicale n’est pas retenue devant la régression spontanée de l’épanchement, l’absence d’aggravation neurologique en plus l’état pathologique de l’os et le diagnostic de pleurésie hémorragique sur descellement de matériel d’ostéosynthèse est retenu après avoir éliminé les autres étiologies.

## Discussion

Les épanchements pleuraux sont le résultat de l’accumulation de liquide dans l’espace pleural en rapport avec une maladie qui peut être pulmonaire, pleurale ou extra-pulmonaire. Ainsi devant une pleurésie unilatérale, la réalisation d’une ponction avec dosage de protides, LDH, pH, coloration de gram et culture est nécessaire, la mesure d’hématocrite est utile pour différencier un hémothorax d’une pleurésie hémorragique [1]. La biopsie pleurale reste un examen utile dans les pays à haute prévalence de tuberculose, bien que la thoracoscopie ait un rendement plus élevé en cas de suspicion de malignité [2]. La présence d’un épanchement hémorragique suggère le plus souvent l’un des trois diagnostics suivant: pathologie maligne, traumatisme, embolie pulmonaire, moins fréquemment une pneumonie, une tuberculose, un trouble hématologique ou une endométriose. Des aspects étiologiques inhabituels des pleurésies hémorragiques sont mentionnés par des publications de plus en plus nombreuses [3, 4]. Les atteintes thoraciques dans le myélome multiple sont présentes dans 46% des cas, cependant la survenue d'un épanchement pleural au cours de l'évolution est rare, retrouvé dans 6% des cas d'une série de 985 cas de myélome, de Kintzer *et al*. [5]. La survie moyenne reste médiocre et dépasse rarement quatre mois à partir de l’infiltration pleurale par les cellules myélomateuses [6-8]. Ce liquide est constamment de type exsudatif [9]. La présence de plasmocytes dans le liquide pleural est nécessaire au diagnostic de pleurésie myélomateuse [9]. Le nombre de cellules est souvent élevé, et doit être associée à la présence de plasmocytes au niveau pleural.

Ce diagnostic a été éliminé chez notre patiente devant l’absence de cellule plasmocytaire et EPP qui était normale. La biopsie pleurale, rarement pratiquée, n'est pas nécessaire au diagnostic et peut être non informante [6, 9]. L'amylose et l'insuffisance cardiaque constituent les causes les plus fréquentes d'épanchement pleural en cas de myélome, les infections et les infarctus pulmonaires sont très rares [5]. Les cas d'épanchement pleuraux myélomateux publiés dans la littérature ne dépassent pas une centaine [9]. Mais était pratiquée chez notre patiente à fin d’éliminer une pleurésie infectieuse notamment tuberculeuse, d’autres pathologies tumorales, une amylose surtout en absence d’autre manifestations. Dans notre observation l’épanchement hémorragique a était rattaché au descellement de la vis d’ostéosynthèse objectivait sur la TDM thoracique, et devant le bilan étiologique qui est revenu négatif, cette complication qui est rare, dont l’hypothèse la plus probable est l’érosion d’un vaisseau dans la cavité pleurale après le descellement de la vis sur le matériel d’ostéosynthèse. Aucun cas similaire dans la littérature n’a été rapporté selon nos connaissances, par contre des complications hémorragiques précoce type hématorachis postopératoire peut survenir dans les 24 à 48 heures suivant le geste chirurgical, il est dû à un saignement le plus souvent d’origine veineuse épidurale, ou parfois à partir d’une tranche de section osseuse. La constitution d’un hématome compressif se manifeste par l’apparition d’une lombalgie intense, puis d’une mono ou d’une poly-radiculalgie déficitaire d’aggravation rapide [10].

## Conclusion

L’intérêt de notre cas c’est de rapporter une complication hémorragique rare secondaire à un descellement d’une vis d’ostéosynthèse dans la cavité pleurale, qui est un diagnostic retenu après avoir éliminé les autres causes de pleurésie hémorragique.
